# Evaluation of anaesthetic protocols for laboratory adult zebrafish (*Danio rerio*)

**DOI:** 10.1371/journal.pone.0197846

**Published:** 2018-05-22

**Authors:** Tânia Martins, Enoque Diniz, Luís M. Félix, Luís Antunes

**Affiliations:** 1 Centre for the Research and Technology of Agro-Environmental and Biological Sciences (CITAB), University of Trás-os-Montes and Alto Douro (UTAD), Vila Real, Portugal; 2 Departamento de Sanidade Animal (DSA), Faculdade de Medicina Veterinária (FMV), Universidade José Eduardo dos Santos (UJES), Huambo, Angola; 3 Institute for Investigation and Innovation in Health (i3S), University of Porto, Porto, Portugal; Washington State University, UNITED STATES

## Abstract

In the last decades, the use of zebrafish (*Danio rerio*) in biomedical research has increased. Anaesthesia is daily used in fish during experimental procedures to avoid discomfort, stress or pain. Also, fish welfare and the reliability of results can be compromised if an unsuitable anaesthetic protocol is used. Therefore, we aimed to refine anaesthetic protocols to be used in adult zebrafish by evaluating the efficacy of different anaesthetics, used alone or in combination. For that, zebrafish were randomly assigned to 8 different groups: 100 μg/mLMS-222 (MS); 0.2 μg/mL etomidate (E); 0.2 μg/mL etomidate + 100 μg/mL lidocaine (E+L); 1.25 μg/mL propofol (P); 1.25 μg/mL propofol + 100 μg/mL lidocaine (P+L); 100 μg/mL ketamine (K); 100 μg/mL ketamine + 1.25 μg/mL medetomidine (K+M); and 100 μg/mL ketamine + 1.25 μg/mL medetomidine/3.125 μg/mL atipamezole (K+M/A). The animals were placed in an anaesthetic water bath, then, the following parameters were registered: time for equilibrium loss and anaesthesia induction, loss of sensitivity to soft and painful stimuli, respiratory rate, recovery time, and activity after recovery. The combined forms of E+L, P+L and K+M were the fastest to induce a surgical anaesthetic stage. Nevertheless, E+L induced respiratory depression, while K+M was shown to have the longer recovery time compared to MS-222, even when atipamezole was added. In conclusion, the P+L combination was shown to provide good anaesthesia with analgesia, without causing a major respiratory depression, providing as well a quick recovery, similar to MS-222.

## Introduction

Laboratory zebrafish (*Danio rerio*) has emerged as a powerful vertebrate model system to study several human diseases [1–3], as well as an important model in toxicological and regeneration studies, among others [4–6]. The several biological and physiological advantages of zebrafish [4, 7], as well as its maintenance costs that are substantially lower than those for mammals [8], have contributed for the increase in the use of this species. Despite this increase, the research on welfare and refinement of procedures in zebrafish is scarce. General anaesthesia is defined as a reversible state resulting in unconsciousness and a total loss of sensation through depression of the central nervous system, and this state may be followed by different levels of analgesia (absence of pain) and muscle relation. The use of an inadequate anaesthetic protocol in zebrafish can cause pain and mortality as well as an increase in the data’s variability during experimental protocols, compromising thus animal welfare as well as scientific outcomes. This can carry a substantial scientific and economic costs in daily research.

Tricaine methanesulfonate or MS-222 is the most used anaesthetic in fish and is considered safe [9]. Although, depending on the dose and exposure times it can have adverse side effects such as aversion, epidermal and corneal lesions, hypoxemia, decreased heart rate and death [10–15]. Such effects can be an issue when deeper stages of anaesthesia and long duration procedures are needed and mainly in small fish such as zebrafish. Besides, MS-222 has been reported to be toxic to humans [16, 17]. As an alternative to MS-222, other anaesthetics have been used or tested, such as etomidate, propofol, lidocaine or ketamine combined with medetomidine.

Etomidate is a potent nonbarbiturate hypnotic drug able to induce a quick anaesthesia induction and recovery. However, this anaesthetic agent is not suitable for major procedures, as it does not induce a surgical anaesthetic stage or analgesia [18, 19].

Propofol is a sedative-hypnotic agent that is capable of anaesthesia induction in a rapid and smooth way and lacks cumulative effects due to its rapid metabolization. Moreover, the recovery from anaesthesia is also quick and complete [20, 21]. Lidocaine hydrochloride, a local anaesthetic, induces anaesthesia quickly and the recovery is also relatively rapid [19], although, in zebrafish, it may present a high variability in the mean times to achieve a given anaesthetic stage or recovery [9]. Our group recently showed that lidocaine combined with propofol induces a quick and safe depth of anaesthesia and may be used as an alternative to MS-222 [20].

Ketamine induces a dissociative anaesthesia with some analgesic component [22, 23], but its use in fish depends on the species. In Salmonid species, it showed excitement, incomplete anaesthesia, apnoea, and prolonged recovery [18]. In zebrafish, ketamine showed neurotoxic effects in larvae [24, 25] and interfered with embryonic development [26]. Medetomidine possesses a sedative action, and provide analgesia and muscle relaxation. This agent is often used in other species in combination with ketamine for its synergistic effects and to oppose the muscle rigidity/twitching induced by ketamine [27]. Moreover, this combination may be reversed by using atipamezole, allowing the control of anaesthesia duration [19].

Due to the wide range of anaesthetic drugs available, protocols for fish anaesthesia usually include only one anaesthetic agent instead of a combination. However, some studies have demonstrated that the mixture of two types of anaesthetic agents can result in a safer and more effective anaesthesia. For instance, the combination of low doses of both isoflurane and MS-222 induced prolonged light anaesthesia time and faster recovery with minimal effects on the heart rate compared with MS-222 alone [11]. Also, Valentim et al. [20] demonstrated that a low dose of propofol was able to achieve a surgical anaesthetic stage (with analgesia) when combined with lidocaine.

Still, no anaesthetic agent is effective in all scenarios. Thus, this study aims to investigate the effects of different anaesthetic agents, used alone or in combination, which could be used as an alternative to MS-222 in order to find an anaesthetic protocol able to provide a better and safer anaesthesia for zebrafish. This is justified due to the current zebrafish importance in biomedical research and the lack of information on the adequacy of anaesthetic protocols used in this species.

## Materials and methods

### Ethics statement

All procedures were carried out under project licence (number: 017216) approved by the National Competent Authority for animal research, named Direção-Geral de Alimentação e Veterinária (DGAV, Lisbon, Portugal). All experimental procedures were performed in accordance with the European Directive 2010/63/EU and with National Law (DL n° 113/2013) on the protection of animals used for scientific purposes, and were locally approved by the University of Trás-os-Montes e Alto Douro and conducted under the scope of a Master’s thesis.

### Animals and housing

Sixty-eight adult AB zebrafish (both male and female) bred in the Animal Facility of the institution were used. Animals were maintained in a 20 L tank at 28 ± 1°C, pH = 6.6–8.5, in a 14 h light /10 h dark cycle, and in an open water system with aeration, and with mechanical and biological filtration. Fish were fed twice a day with a commercial diet (Sera, Heinsberg, Germany) supplemented with artemia. Fish were randomly assigned to 8 experimental groups, composed of 8 fish each. A control group, not anaesthetised, was composed of 4 fish. For the recovery after anaesthesia, fish were placed in a 5 L tank for 48 h and then transferred to a 20 L tank. All tanks had UV sterilized water.

### Anaesthetic agents

The following drugs were used in the present study: Etomidate (2 mg/mL, Lipuro, B. Braun, Melsungen, Germany); Propofol (10 mg/mL, Lipuro, Braun VetCare SA, Barcelona, Spain); Lidocaine (20 mg/mL, B.Braun Medical, Queluz de Baixo, Barcarena, Portugal); Ketamine (1 g/mL, Clorketam 1000, Vetoquinol S.A., Lure, France); Medetomidine (1 mg/mL, Domtor, Orion Corporation Orioninte, Espoo, Finland); Atipamezole (5 mg/mL, Antisedan, Orion Corporation Orioninte, Espoo, Finland). The buffered MS-222 solution was prepared by adding ethyl 3-amino-benzoate methanesulfonate powder (Sigma-Aldrich, Sintra, Portugal) to system water, making a stock solution of 1.5 g/L buffered with sodium bicarbonate until pH reached 7.2–7.4. The anaesthetic protocols with the final concentrations tested in this study are presented in Table 1.

**Table 1:** Anaesthetic protocols.

Single anaesthetic agent	Combination of anaesthetic agents
100 μg/mL MS-222 (MS)	2 μg/mL E + 100 μg/mL Lidocaine (L)
2 μg/mL Etomidate (E)	1.25 μg/mL P + 100 μg/mL L
1.25 μg/mL Propofol (P)	100 μg/mL K + 1.25 μg/mL Medetomidine (M)
100 μg/mL Ketamine (K)	100 μg/mL K + 1.25 μg/mL M / 3.125 μg/mL Atipamezole (A)

### Anaesthesia and post-anaesthetic recovery assessment

All the anaesthetic solutions were prepared in a beaker of 200 mL with a UV water sterilized at 28 ± 1°C and pH = 6.6–8.5. The anaesthetics were added individually to the proper volume of water in the beaker and then stirred. Some anaesthetics presented different colours in solution, allowing their identification, which prevented the researcher from being blind to the treatment of each fish. For that reason, to allow an equal approach to the different drugs, it was decided to know which drugs were being used during all the trials. Although some anaesthetic solutions presented a colouration, they were sufficiently transparent to allow animal visualization.

Immediately after the anaesthetic solution was homogenized, zebrafish were placed in the anaesthetic water bath, and the time count was initiated to measure the loss of the equilibrium, time for induction, and the loss of sensitivity to soft and painful stimuli. The anaesthetic parameters were assessed as previously described [20] with slight modifications. Briefly, loss of equilibrium was considered at the time of the change from ventral to dorsal decubitus (lying down) position. The time of anaesthetic induction was measured from the immersion point until the fish no longer moved, and remained in a total and permanent imbalance in the bottom of the beaker in dorsal decubitus. The response to a soft stimulus was assessed by touching the lateral side of the fish with a plastic pipette, while the response to a painful stimulus, a tail pinch, was evaluated by gently pressing the caudal fin with forceps. The response to the soft or painful stimuli was tested every 10 sec. The respiratory rate was measured after equilibrium loss and after the loss of response to a soft touch, by counting opercular movements during 1 min.

After the loss of the tail pinch reflex, the animal was placed alone in a tank to recover. Then, the time the fish took to start moving and to recover the equilibrium, adopting a ventral position, was recorded. After the full recovery, zebrafish activity was evaluated 5 h and 24 h post-anaesthesia (hpa). The frequency of crossing a longitudinal line in the tank per minute (crossings/min) was used to determine fish activity. For the control group, the animals were distributed separately in small tanks and kept in rest for 5 h for their habituation, only then their activity was assessed. Fish were kept in visual contact with other zebrafish to minimize the isolation for activity assessment. After 48 h of recovery, the animals were collected individually and taken to the larger tank prepared to accommodate all the fish already submitted to the anaesthetic protocols.

### Statistical analysis

Statistical analysis of the data was done using GraphPad Prism (GraphPad Software). First, to access if data followed a Gaussian distribution, anaesthesia and behaviour data were analysed for normality by using D'Agostino-Pearson normality test. Then, according to the normality test results, parametric or non-parametric tests were applied. For the data following a Gaussian distribution, namely equilibrium loss data, statistical significance was assessed with the one-way ANOVA parametric test followed by Dunnett's multiple-comparison test to compare the effect of different anaesthetic protocols with MS-222. For the data that did not follow a Gaussian distribution, namely all other parameter´s data, the statistical significance was evaluated using the non-parametric Kruskal-Wallis test (non-parametric ANOVA) followed by Dunn's multiple comparisons test to compare the effect of different anaesthetic protocols with MS-222. Values of p<0.05 were considered statistically significant. Although there are parametric and non-parametric data, all data in graphs are presented as median [interquartile range]. The interquartile range is defined as the difference between the first quartile (25th percentile) and the third quartile (75th percentile) of an ordered range of data.

## Results

All animals of all treatment groups lost all the reflexes analysed and no mortality occurred during or after anaesthesia. The anaesthetic protocols tested did not induce distressful behaviours in fish during induction or recovery, except for ketamine alone, in which, before reaching the induction phase, fish underwent phases of excitation with erratic, accelerated and intense movements in almost all directions, with the predominance of circular movements. Given that MS-222 is the most used anaesthetic agent in fish, the anaesthetic parameters obtained for MS-222 were used for comparison with the outcomes of the other anaesthetic protocols.

**Fig 1:**
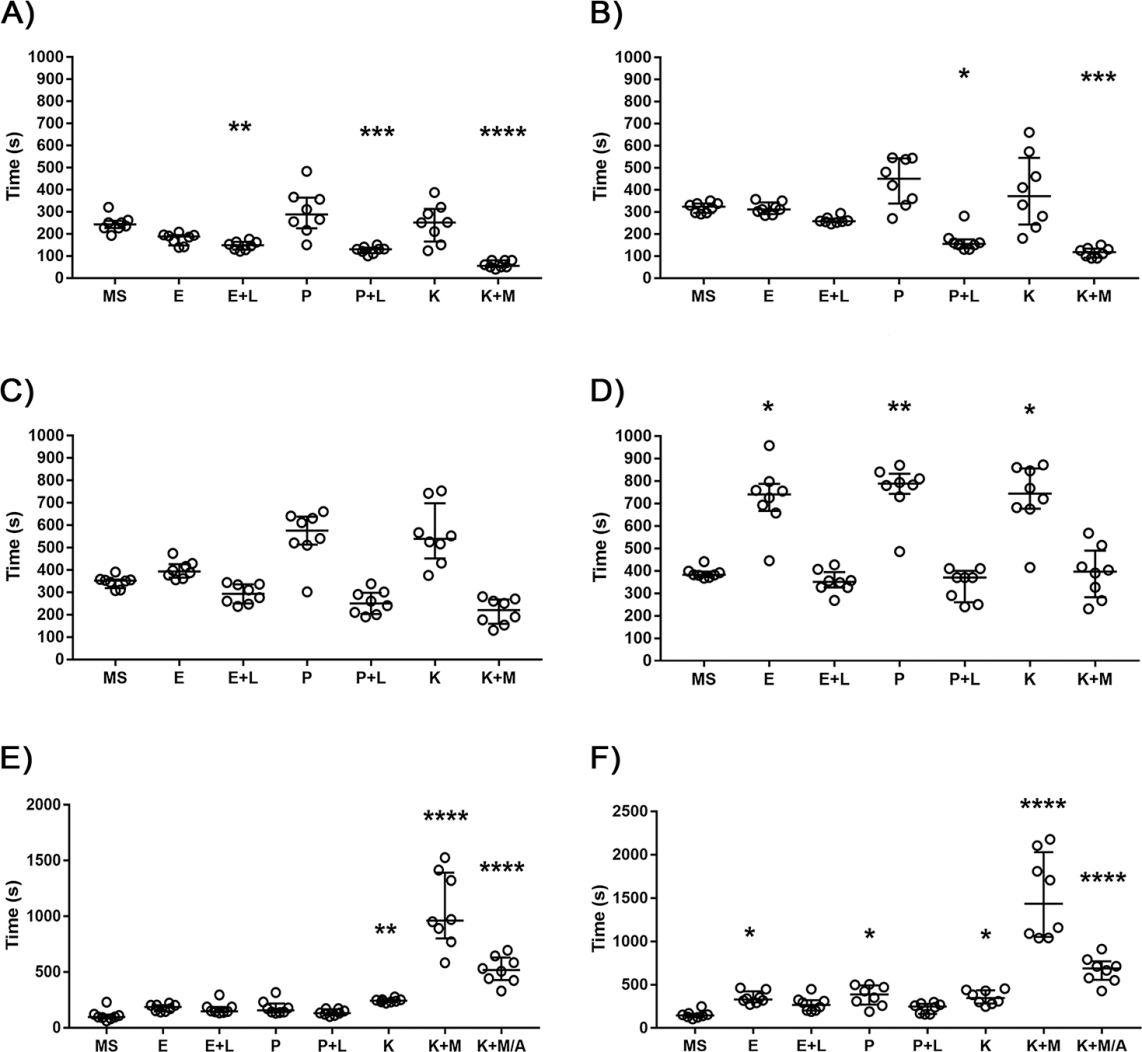
Anaesthetic parameters of adult zebrafish when treated with different anaesthetic protocols. A) Equilibrium loss, B) anaesthesia induction, C) loss of reaction to a soft stimulus, D) loss of reaction to a painful stimulus, E) initiation of the movements, and F) equilibrium recovery of adult zebrafish after being subjected to an anaesthetic bath of: 100 μg/mL MS-222 (MS), 0.2 μg/mL etomidate (E); 0.2 μg/mL etomidate + 100 μg/mL lidocaine (E+L); 1.25 μg/mL propofol (P); 1.25 μg/mL propofol + 100 μg/mL lidocaine (P+L); 100 μg/mL ketamine (K); 100 μg/mL ketamine + 1.25 μg/mL medetomidine (K+M); or 100 μg/mL ketamine + 1.25 μg/mL medetomidine / 3.125 μg/mL atipamezole (K+M/A). Each point represents an animal (n = 8). Data are expressed as median [interquartile range]. *p<0.05, **p<0.01, ***p<0.001, ****p<0.0001 when compared with MS-222. One-way ANOVA parametric test followed by Dunnett's multiple-comparison test was used for equilibrium loss analysis, nevertheless, data are expressed as median [interquartile range]. Non-parametric Kruskal-Wallis test followed by Dunn's multiple comparisons test was used for the analysis of all other parameters.

Regarding the time for equilibrium loss (Fig 1A; S1 Table), comparing to the median of MS, the treatments with E+L (p<0.01), P+L (p<0.001) and K+M (p<0.0001) induced the loss of equilibrium more quickly, while the protocols with E, P and K were not statistically different from MS. Concerning the time for anaesthesia induction (Fig 1B; S1 Table), only the treatments with P+L (p<0.05) and K+M (p<0.001) were significantly quicker than MS to induce anaesthesia. The protocols with E, E+L, P and K were not statistically different from MS. No significant differences were found between MS and all the other treatment groups regarding the loss of reaction to a soft stimulus (Fig 1C; S1 Table), although P and K showed a tendency to take longer to lost this reflex. Regarding loss of reaction to a painful stimulus (Fig 1D; S1 Table), no significant differences were found between MS and the treatment groups E+L, P+L and K+M. By the opposite, the treatments with E (p<0.05), P (p<0.01) and K (p<0.05) took longer to induce loss of response to a painful stimulus comparing to MS. In the assessment of the recovery phase, two parameters were registered: the initiation of the movements and the recovery of the equilibrium. Concerning the initiation of the movements (Fig 1E; S2 Table), the K (p<0.01), K+M (p<0.0001) and K+M/A (p<0.0001) groups took longer time for movements to begin compared to MS, while E, E+L, P and P+L groups showed no significant differences compared to MS. As regards the time for the recovery of the equilibrium (Fig 1F; S2 Table), no significant differences were found between MS and the E+L and P+L groups, while the treatments with E (p<0.05), P (p<0.05), K (p<0.05), K+M (p<0.0001) and K+M/A (p<0.0001) took longer time to recover the equilibrium.

**Fig 2:**
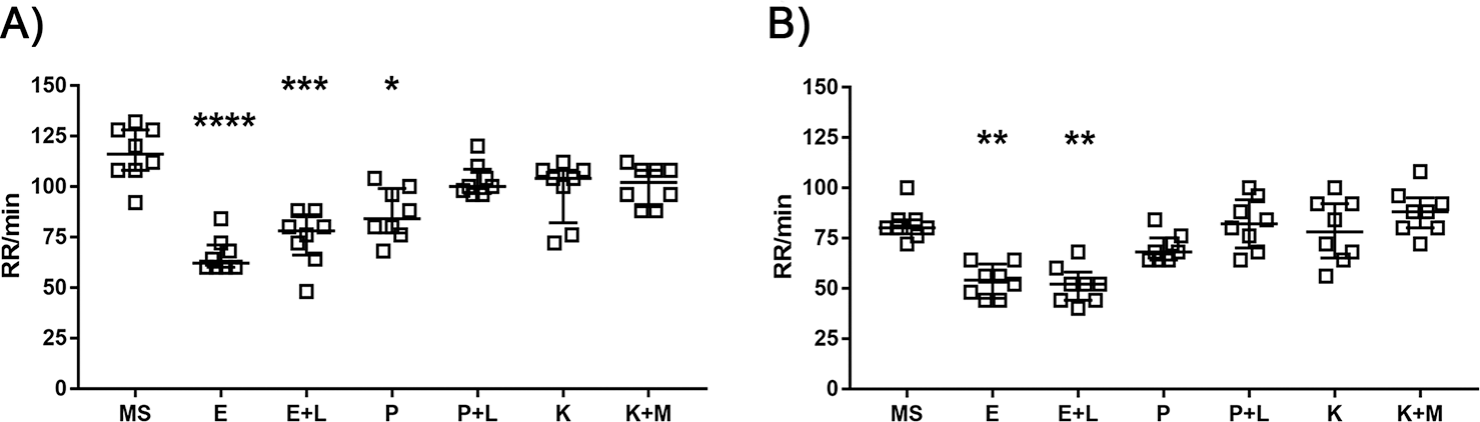
Respiratory rate (RR) per minute of adult zebrafish when treated with different anaesthetic protocols. A) RR after loss of equilibrium and B) RR after loss of soft stimulus response of adult zebrafish after being subjected to an anaesthetic bath of: 100 μg/mL MS-222 (MS), 0.2 μg/mL etomidate (E); 0.2 μg/mL etomidate + 100 μg/mL lidocaine (E+L); 1.25 μg/mL propofol (P); 1.25 μg/mL propofol + 100 μg/mL lidocaine (P+L); 100 μg/mL ketamine (K); 100 μg/mL ketamine + 1.25 μg/mL medetomidine (K+M); or 100 μg/mL ketamine + 1.25 μg/mL medetomidine / 3.125 μg/mL atipamezole (K+M/A). Each point represents an animal (n = 8). Data are expressed as median [interquartile range]. *p<0.05, **p<0.01, ***p<0.001, ****p<0.0001 when compared with MS-222. Non-parametric Kruskal-Wallis test followed by Dunn's multiple comparisons test.

Concerning the respiratory rate (RR) after loss of equilibrium (Fig 2A; S3 Table), the treatments with E (p<0.0001), E+L (p<0.001), and P (p<0.05) significantly lowered the opercular movements per minute comparing to MS, while P+L, K and K+M groups showed no significant differences comparing to MS. In a similar way, the treatments with E (p<0.01) and E+L (p<0.01) significantly lowered the respiratory rate after the loss of the soft stimulus reflex (Fig 2B; S3 Table) comparing to MS, while no significant differences were found between MS and the rest of the treatment groups.

**Fig 3:**
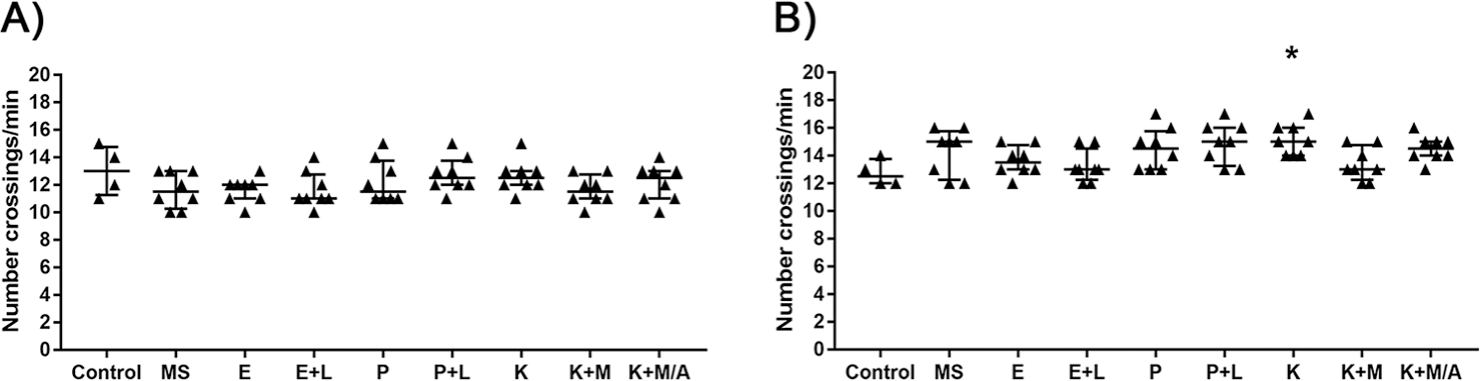
Number of crossings per minute of adult zebrafish through the longitudinal line of the tank after being treated with different anaesthetic protocols. The activity of zebrafish was analysed at A) 5 h and B) 24 h after being subjected to the following anaesthetic baths: 100 μg/mL MS-222 (MS), 0.2 μg/mL etomidate (E); 0.2 μg/mL etomidate + 100 μg/mL lidocaine (E+L); 1.25 μg/mL propofol (P); 1.25 μg/mL propofol + 100 μg/mL lidocaine (P+L); 100 μg/mL ketamine (K); 100 μg/mL ketamine + 1.25 μg/mL medetomidine (K+M); or 100 μg/mL ketamine + 1.25 μg/mL medetomidine / 3.125 μg/mL atipamezole (K+M/A). A group of non-anaesthetized animals served as Control. Each point represents an animal (n = 4 for control group; n = 8 for all other groups). Data are expressed as median [interquartile range]. *p<0.05 when compared with Control. Non-parametric Kruskal-Wallis test followed by Dunn's multiple comparisons test.

In the assessment of activity 5 hpa (Fig 3A; S4 Table), no significant differences were found between the Control and the treatment groups. As regards the activity 24 hpa (Fig 3B; S4 Table), only K (p<0.05) group showed a slightly significant increase in the number of crossings comparing to Control, while the other treatments showed no significant differences comparing to Control.

## Discussion

The use of zebrafish in biomedical research has increased in the last decades. When fish are submitted to minor or major procedures, analgesia and anaesthesia must be provided. MS-222 is the most common anaesthetic agent used in fish species, including zebrafish [28], nevertheless, it can cause adverse side effects, compromising animal welfare as well as scientific outcomes. In the present study, we tested several immersion anaesthetic protocols, using isolated drugs or in combination, and compare their efficacy with that of MS-222 in adult zebrafish to investigate if there are safer and more efficient alternatives than the MS-222. We found that the anaesthetic agents tested alone showed to take higher time to achieve the analysed anaesthetic parameters, showing also a greater interindividual variability. The combinations of etomidate/lidocaine, propofol/lidocaine, or ketamine/medetomidine were able to induce loss of equilibrium in less than 3 min, while ketamine/medetomidine registered the lower values, less than 1 min. Also, the combinations propofol/lidocaine and ketamine/medetomidine were significantly faster than MS-222 to induce anaesthesia. These results showed that drugs in combination can be more efficient in sedation and anaesthesia induction. Nevertheless, etomidate/lidocaine combination induced respiratory depression. Also, ketamine/medetomidine combination was shown to have the longer recovery time (within 24 min) comparing to MS-222 (within 2.5 min), even when atipamezole was added to reverse medetomidine (within 11.5 min), recovery was slower than with MS-222. These data suggest that these combinations may not be suitable for major procedures that require longer exposure times, but may be used for quick procedures that need just rapid immobilization, such as handling and tagging. On the contrary, the combination propofol/lidocaine provided a quick and good anaesthesia with analgesia, without causing a major respiratory depression, providing as well a quick recovery in less than 4.2 min. These data suggest that the combination propofol/lidocaine is suitable for sedation, minor and major surgical procedures in zebrafish.

Etomidate, a potent non-barbiturate hypnotic drug, is used as an immersion anaesthetic in fish, although it does not have an analgesic effect [19]. In the present study, etomidate induced light anaesthesia with no significant differences from MS-222, followed by a pronounced respiratory depression. Etomidate also induced the loss of reaction to a painful stimulus, however, it was significantly slower than MS to achieve this stage, presenting a great variability between individuals, suggesting that etomidate is not suitable for major procedures that require a surgical plane of anaesthesia. Also, Amend et al demonstrated that, in zebrafish, etomidate alone induced light anaesthesia (the reaction to a painful stimulus was not explored), and could cause mortality depending on the dose or time of exposure, i.e. exposure to 3 mg/L etomidate for 10 min resulted in 25% of mortality, while exposure for 20 min resulted in 100% mortality [29]. In the present study, no mortality was registered with this anaesthetic agent, probably because the dose used and the exposure time to etomidate were much lower than the doses and exposure times tested in the study of Amend et al. [29]. The combination of two types of anaesthetic drugs can result in a safer and more efficient anaesthesia [11, 20], since it will allow a reduction in dose and consequently in side effects and mortality, providing also a better recovery [30]. In this regard, lidocaine is a local anaesthetic with an analgesic effect in rainbow trout (*Oncorhynchus mykiss*) and zebrafish [28, 30], and with the ability to induce anaesthesia in medaka (*Oryzias dancena*) with no side-effects [31]. Lidocaine can also induce a surgical plane of anaesthesia in zebrafish within 50 seconds (with high variability) when exposed to 325 mg/L lidocaine, but it could cause mortality in fish exposed to 350 mg/L [9]. In the present study, when etomidate was combined with lidocaine, the time needed for zebrafish to achieve a surgical stage of anaesthesia was decreased, and the time for equilibrium recovery was not significantly different from MS-222, contrary to etomidate used alone, which was higher. Nevertheless, the combination etomidate/lidocaine also showed a lower respiratory rate comparing to MS-222, suggesting that it is not suitable for long procedures.

Propofol is a short-acting hypnotic drug that produces a rapid anaesthesia induction with short duration being rapidly metabolized and without cumulative effects, being also capable of causing cardiorespiratory depression [32]. In spotted bamboo shark (*Chylloscyllium plagiosum*), the administration of propofol, intravenously, was considered safe, as it induced a satisfactory plane of anaesthesia and did not affect the heart and respiratory rate [33], while in Gulf of Mexico sturgeon (*Acipenser oxyrinchus de soti*) mild bradycardia and bradypnea occurred [32]. Propofol has also been administered by immersion in smaller fish such as rainbow trout (*Oncorhynchus mykiss*) [34], goldfish (*Carassius auratus*) [21] and zebrafish [20, 35]. In the present study, propofol alone showed some interindividual variability in several parameters analysed, namely during anaesthesia induction until reaching the surgical plane of anaesthesia and in the equilibrium recovery. When lidocaine was combined, the interindividual variability decreased. Also, this combination induced equilibrium loss and anaesthesia faster than MS-222. In addition, while propofol alone was significantly slower than MS-222 to induce loss of reaction to a painful stimulus, the combination with lidocaine showed no differences from MS-222, and the same was observed for equilibrium recovery. Moreover, propofol alone reduced significantly the respiratory rate after the loss of equilibrium comparing to MS-222, while the combination with lidocaine showed no statistical differences from MS-222. These data suggest that the combination propofol/lidocaine can be used for sedation, as well as for minor and major surgical short procedures in zebrafish, being an alternative to MS-222. Our group has recently tested several doses of propofol alone or in combination with lidocaine in adult zebrafish, showing that only the protocols with a combination of drugs were able to induce deep anaesthesia, besides, mortality was observed in several protocols[20]. In the present study, the combination propofol/lidocaine was also able to decrease the time to achieve the different anaesthetic parameters. Moreover, propofol alone was able to induce full anaesthesia, but the fish took more than 5 min to loss the response to the tail pinch. A lower dose of propofol was used in the present study compared to the study of Valentim et al. [20], which could explain the lack or mortality observed.

Ketamine is an anaesthetic agent that induces a dissociative anaesthesia with some analgesia and its use is not recommended alone in major surgical procedures [36]. In zebrafish, sub-anaesthetic doses of ketamine were shown to induce anxiolytic-like effects, hyperactivity, to increase circling behaviour, impaired habituation, and reduce shoaling [37, 38]. In the present study, when ketamine alone was used, before reaching the induction phase, zebrafish underwent phases of excitation with erratic, accelerated and intense movements in almost all directions, with the predominance of circular movements. Also, ketamine alone showed a high interindividual variability in several parameters analysed, from equilibrium loss to loss of response to a painful stimulus. This variability may be explained by the interindividual differences in hepatic clearance and ketamine pharmacokinetics [39]. Ketamine was not significantly different from MS-222, except in the loss of response to a painful stimulus, in the beginning of movements and equilibrium recovery parameters, in which it was significantly slower than MS-222 to achieve those stages. Medetomidine has been used in mammals to induce sedation, analgesia, and anaesthesia, and its effects can be reversed with atipamezole. Therefore, the combination of ketamine with medetomidine is used to induce a general anaesthesia with good sedative and analgesic qualities, with a wide safety margin, that can be reversed with atipamezole [36]. In this study, ketamine combined with medetomidine was the fastest to induce loss of equilibrium and anaesthesia, nevertheless, it showed the longer recovery time, even when atipamezole was administered to reverse medetomidine effects. In bonito (*Sarda chiliensis*) and Pacific mackerel (*Scomber japonica*), intramuscularly anaesthetised with ketamine/medetomidine, the administration of atipamezole at a dose of five times the dose of medetomidine has been shown to be effective in reversing anaesthesia [40]. In the present study, the dose of atipamezole used was only three times the dose of medetomidine, which may justify the fact that it was not sufficient to completely reverse medetomidine effects in order to induce a faster recovery.

Regarding the recovery phase, except for etomidate/lidocaine and propofol/lidocaine combinations, all protocols were significantly slower than MS-222 in providing full equilibrium recovery. This was particularly observed with the combination ketamine/medetomidine and even if atipamezole was used. Despite this, at 5 h and 24 h post-anaesthesia, the activity of zebrafish was completely recovered in all protocols without significant differences from the non-anaesthetized Control group, except for ketamine alone which showed a slight increase in the activity at 24 h post-anaesthesia. Valentim et al. [20] also showed that in zebrafish subjected to several doses of propofol or propofol/lidocaine combinations, and MS-222, all had their activity recovered at 1 h, 5 h and 24 h post-anaesthesia.

Besides the quality of a given anaesthetic for a particular procedure, other factors such as the availability, handling, preparation, storage and price, can influence the choice of the anaesthetic for a given situation. MS-222 is available as a powder and is soluble in water, however, due to its acidic nature, the anaesthetic bath needs to be buffered. MS-222 stock solutions of 10% can be prepared, which are stable and effective for up to 3 months if kept in a dark and cool place [13, 19]. Also, MS-222 is not licensed in some European countries [13], which reduces its availability. Regarding the other anaesthetic agents tested in this study, their presentation in a solution or as an emulsion, in the case of propofol and etomidate, facilitates their direct use, without the need to prepare a stock solution. According to manufacturers, their shelf life can be around 2 years, although, it is advised to be used within one month after opening, or immediately in the case of propofol or etomidate. Regarding the costs, Tricaine Pharmaq 1000 mg/g (MS-222) is the most expensive per package (100 g), followed by medetomidine, atipamezole and ketamine (10 mL per flask for each). Then follows Etomidate Lipuro at 2 mg/mL (10 mL per ampoule), and Propofol Lipuro 2% (50 mL per flask) and Lidocaine 2% (10 mL per ampoule). Thus, the choice of an anaesthetic or combination of anaesthetics may be influenced not only by the number and nature of the procedures but also by the amount of anaesthetic required to avoid waste.

In conclusion, all the protocols tested were able to induce anaesthesia, but only the protocols in combination appear to be suitable to induce a surgical plane of anaesthesia similar to that of MS-222. Although none of the protocols tested seemed to be better than MS-222, this work shows that there are some anaesthetic protocols, such as propofol/lidocaine that can be as efficient as MS-222, and so can be used as an alternative. The combination of propofol with lidocaine demonstrated to be the best from all the protocols tested, since it provided good anaesthesia with analgesia, without causing a major respiratory depression, providing as well a quick recovery, being suitable for sedation and short surgical procedures in zebrafish. The overall outcomes of propofol/lidocaine were similar to those of MS-222. This anaesthetic protocol may have advantages over MS-222 since it does not require a special preparation such as solution buffering. Nevertheless, the appropriate anaesthetic protocol should always be chosen based on the purpose of the experimental procedure. Furthermore, biochemical analyses were not performed in order to evaluate biomarkers of stress or other physiological changes to attest these protocols safety in zebrafish. The pharmacokinetics of anaesthetics was not explored in the current study, therefore, the evaluation of representative durations of the surgical anaesthetic state, as well as the consequent effects of long exposure times for each protocol, should be taken in consideration for future studies. Also, the evaluation of multiple exposure effects to the same anaesthetic would be important. Nevertheless, the protocols tested seemed to cause no distressful behaviours, except for ketamine used alone, nor mortality, making them appear safe to zebrafish.

## Supporting information

S1 Table Time for equilibrium loss, anaesthesia induction, loss of reaction to a soft stimulus, and loss of reaction to a painful stimulus, for the different protocols tested, in seconds (s), median [interquartile range]. (PDF)

S2 Table Time for the initiation of the movements and recovery of the equilibrium, for the different protocols tested, in seconds (s), median [interquartile range]. (PDF)

S3 Table Respiratory rate (RR) after loss of equilibrium and loss of the soft stimulus reflex, for the different protocols tested, in RR per minute (RR/min), median [interquartile range]. (PDF)

S4 Table Activity 5 hours and 24 hours post-anaesthesia, for the different protocols tested, in number of crosses per minute (cross/min), median [interquartile range]. (PDF)
