# Supplementary material for: Evaluation of anaesthetic protocols for laboratory adult zebrafish (Danio rerio)
Source: PLoS One. 2018 May 22;13(5):e0197846. doi: 10.1371/journal.pone.0197846 (PMC5963751; doi:10.1371/journal.pone.0197846)
Supplement: S1 Table — (PDF) [file pone.0197846.s001.pdf]

S1 Table. Time for equilibrium loss, anaesthesia induction, loss of reaction to a soft stimulus, and loss of reaction to a painful stimulus, for the different protocols tested, in seconds (s), median [interquartile range].

Anaesthetic protocols	Time for equilibrium loss	Time for anaesthesia	Time for loss of reaction to a soft stimulus	Time for loss of reaction to a painful stimulus
**100 µg/mL MS**	242.5 s [214.7 to 276.1 s]	324 s [300 to 338.7]	352 s [323.9 to 368.6 s]	382.5 s [370.2 to 408.8 s]
**2 µg/mL E**	187.5 s [155.8 to 198.9 s]	311 s [292.8 to 337.7 s]	393 s [367.1 to 432.4 s]	740 s [603.3 to 844.4 s]
**2 µg/mL E + 100 µg/mL L**	149 s [130.9 to 163.9 s]	257.5 s [249 to 274.5 s]	293 s [256.9 to 329.1 s]	351.5 s [310.9 to 393.1 s]
**1.25 µg/mL P**	287.5 s [214.2 to 386 s]	450 s [346.1 to 525.6 s]	575.5 s [454.5 to 648.7 s]	788.5 s [662.2 to 861.1 s]
**1.25 µg/mL P + 100 µg/mL L**	130 s [113 to 139.8 s]	155 s [126.9 to 208.3 s]	250 s [209.2 to 298 s]	370 s [281.3 to 396.2 s]
**100 µg/mL K**	251 s [175.3 to 320.4 s]	371 s [250.4 to 530.8 s]	538.5 s [445.8 to 669 s]	744 s [604.7 to 854.6 s]
**100 µg/mL K + 1.25 µg/mL M**	55 s [47.52 to 74.98 s]	117.5 s [97.72 to 134.8 s]	220 s [165.4 to 262.3 s]	397 s [294.1 to 485.9 s]

MS – MS-222; E – Etomidate; L – Lidocaine; P – Propofol; K – Ketamine; M – Medetomidine.
