# Supplementary material for: Evaluation of anaesthetic protocols for laboratory adult zebrafish (Danio rerio)
Source: PLoS One. 2018 May 22;13(5):e0197846. doi: 10.1371/journal.pone.0197846 (PMC5963751; doi:10.1371/journal.pone.0197846)
Supplement: S2 Table — (PDF) [file pone.0197846.s002.pdf]

S2 Table. Time for the initiation of the movements and recovery of the equilibrium, for the different protocols tested, in seconds (s), median [interquartile range].

Anaesthetic protocols	Time for the initiation of the movements	Time for the recovery of the equilibrium
**100 µg/mL MS**	95 s [66.76 to 152.7 s]	147.5 s [117.2 to 189.6 s]
**2 µg/mL E**	185 s [153.5 to 204.5 s]	330.5 s [292.1 to 408.9 s]
**2 µg/mL E + 100 µg/mL L**	148 s [127 to 216.3 s]	268 s [204.9 to 350.3 s]
**1.25 µg/mL P**	155 s [126.2 to 233 s]	388.5 s [278.2 to 475.3 s]
**1.25 µg/mL P + 100 µg/mL L**	131 s [113.4 to 157.1 s]	250 s [181.8 to 278.7 s]
**100 µg/mL K**	243 s [228.7 to 257.3 s]	347.5 s [288.9 to 423.6 s]
**100 µg/mL K + 1.25 µg/mL M**	960 s [775.6 to 1330 s]	1434 s [1108 to 1925 s]
**100 µg/mL K + 1.25 µg/mL M / 3.125 µg/mL A**	518 s [417.9 to 619.9 s]	689 s [543.3 to 794.2 s]

MS – MS-222; E – Etomidate; L – Lidocaine; P – Propofol; K – Ketamine; M – Medetomidine; A – Atipamezole.
