# Supplementary material for: Evaluation of anaesthetic protocols for laboratory adult zebrafish (Danio rerio)
Source: PLoS One. 2018 May 22;13(5):e0197846. doi: 10.1371/journal.pone.0197846 (PMC5963751; doi:10.1371/journal.pone.0197846)
Supplement: S3 Table — (PDF) [file pone.0197846.s003.pdf]

S3 Table. Respiratory rate (RR) after loss of equilibrium and loss of the soft stimulus reflex, for the different protocols tested, in RR per minute (RR/min), median [interquartile range].

Anaesthetic protocols	RR after loss of equilibrium	RR after the loss of the soft stimulus reflex
**100 µg/mL MS**	116 RR/min [104.7 to 127.3 RR/min]	80 RR/min [75.08 to 88.92 RR/min]
**2 µg/mL E**	62 RR/min [58.85 to 73.15 RR/min]	54 RR/min [46.83 to 60.17 RR/min]
**2 µg/mL E + 100 µg/mL L**	78 RR/min [63.35 to 85.65 RR/min]	52 RR/min [43.82 to 59.18 RR/min]
**1.25 µg/mL P**	84 RR/min [75.93 to 97.07 RR/min]	68 RR/min [64.07 to 75.93 RR/min]
**1.25 µg/mL P + 100 µg/mL L**	100 RR/min [96.08 to 109.9 RR/min]	82 RR/min [71.43 to 92.57 RR/min]
**100 µg/mL K**	104 RR/min [85.23 to 110.8 RR/min]	78 RR/min [65.37 to 91.63 RR/min]
**100 µg/mL K + 1.25 µg/mL M**	102 RR/min [92.42 to 108.6 RR/min]	88 RR/min [78.71 to 97.29 RR/min]

MS – MS-222; E – Etomidate; L – Lidocaine; P – Propofol; K – Ketamine; M – Medetomidine.
