# Supplementary material for: Evaluation of anaesthetic protocols for laboratory adult zebrafish (Danio rerio)
Source: PLoS One. 2018 May 22;13(5):e0197846. doi: 10.1371/journal.pone.0197846 (PMC5963751; doi:10.1371/journal.pone.0197846)
Supplement: S4 Table — (PDF) [file pone.0197846.s004.pdf]

S4 Table. Activity 5 hours and 24 hours post-anaesthesia, for the different protocols tested, in number of crosses per minute (cross/min), median [interquartile range].

Anaesthetic protocols	Activity 5 hours post-anaesthesia	Activity 24 hours post-anaesthesia
**Control**	13 cross/min [10.09 to 15.91 cross/min]	12.5 cross/min [11.23 to 14.27 cross/min]
**100 µg/mL MS**	11.5 cross/min [10.54 to 12.71 cross/min]	15 cross/min [12.85 to 15.65 cross/min]
**2 µg/mL E**	12 cross/min [10.86 to 12.39 cross/min]	13.5 cross/min [12.74 to 14.51 cross/min]
**2 µg/mL E + 100 µg/mL L**	11 cross/min [10.54 to 12.71 cross/min]	13 cross/min [12.28 to 14.22 cross/min]
**1.25 µg/mL P**	11.5 cross/min [10.93 to 13.57 cross/min]	14.5 cross/min [13.24 to 15.76 cross/min]
**1.25 µg/mL P + 100 µg/mL L**	12.5 cross/min [11.68 to 13.82 cross/min]	15 cross/min [13.66 to 16.09 cross/min]
**100 µg/mL K**	12.5 cross/min [11.63 to 13.62 cross/min]	15 cross/min [14.18 to 16.07 cross/min]
**100 µg/mL K + 1.25 µg/mL M**	11.5 cross/min [10.74 to 12.51 cross/min]	13 cross/min [12.38 to 14.37 cross/min]
**100 µg/mL K + 1.25 µg/mL M / 3.125 µg/mL A**	12.5 cross/min [10.99 to 13.26 cross/min]	14.5 cross/min [13.73 to 15.27 cross/min]

MS – MS-222; E – Etomidate; L – Lidocaine; P – Propofol; K – Ketamine; M – Medetomidine; A – Atipamezole.
